# Relationship between Speech Production and Perception in People Who Stutter

**DOI:** 10.3389/fnhum.2016.00224

**Published:** 2016-05-18

**Authors:** Chunming Lu, Yuhang Long, Lifen Zheng, Guang Shi, Li Liu, Guosheng Ding, Peter Howell

**Affiliations:** ^1^State Key Laboratory of Cognitive Neuroscience and Learning & IDG/McGovern Institute for Brain Research, Beijing Normal UniversityBeijing, China; ^2^Center for Collaboration and Innovation in Brain and Learning Sciences, Beijing Normal UniversityBeijing, China; ^3^Division of Psychology and Language Sciences, University College LondonLondon, UK

**Keywords:** speech perception, speech production, stuttering, causal connection, motor area

## Abstract

Speech production difficulties are apparent in people who stutter (PWS). PWS also have difficulties in speech perception compared to controls. It is unclear whether the speech perception difficulties in PWS are independent of, or related to, their speech production difficulties. To investigate this issue, functional MRI data were collected on 13 PWS and 13 controls whilst the participants performed a speech production task and a speech perception task. PWS performed poorer than controls in the perception task and the poorer performance was associated with a functional activity difference in the left anterior insula (part of the speech motor area) compared to controls. PWS also showed a functional activity difference in this and the surrounding area [left inferior frontal cortex (IFC)/anterior insula] in the production task compared to controls. Conjunction analysis showed that the functional activity differences between PWS and controls in the left IFC/anterior insula coincided across the perception and production tasks. Furthermore, Granger Causality Analysis on the resting-state fMRI data of the participants showed that the causal connection from the left IFC/anterior insula to an area in the left primary auditory cortex (Heschl’s gyrus) differed significantly between PWS and controls. The strength of this connection correlated significantly with performance in the perception task. These results suggest that speech perception difficulties in PWS are associated with anomalous functional activity in the speech motor area, and the altered functional connectivity from this area to the auditory area plays a role in the speech perception difficulties of PWS.

## Introduction

Speech and general motor programming deficits have both been reported in people who stutter (PWS) (Fox et al., 1996; Stager et al., 2005; De Nil et al., 2008; Lu et al., 2010a; Smith et al., 2012; Smits-Bandstra and De Nil, 2013; Smits-Bandstra and Gracco, 2015). With respect to speech deficits, speech production difficulties are apparent in PWS and are associated with anomalous neural functional activity in various brain areas (Fox et al., 1996; Braun et al., 1997; Ingham et al., 2000; Stager et al., 2003; De Nil et al., 2008; Watkins et al., 2008; Chang et al., 2009; Kell et al., 2009; Lu et al., 2009, 2010b; Jiang et al., 2012; Cai et al., 2014; Belyk et al., 2015). PWS and controls also show behavioral and neural functional activity differences during speech *perception* (Weber-Fox et al., 2008; Liotti et al., 2010; Sato et al., 2011; Jansson-Verkasalo et al., 2014; Pelczarski and Yaruss, 2014). For example, children who stutter (CWS) have poorer performance on sound elision and blending tasks compared to peer controls (Pelczarski and Yaruss, 2014). Further, CWS do not show significant Mismatch Negativity amplitude in EEG potentials to syllables that have linguistic features that deviate from normal ones (Jansson-Verkasalo et al., 2014). In addition, both adults and children who stutter do not show the expected left lateralized hemodynamic response when two aurally presented nonsense syllables that differ by one phoneme are compared (Sato et al., 2011). It is not known, however, whether such anomalous neural activity during speech perception for PWS is independent of, or related to, that seen in speech production.

A relationship between speech perception and production is supported by brain imaging evidence that shows that speech perception activates the left inferior frontal cortex (IFC), insula, and pre/primary motor cortex (PMC) which are all involved in the control of articulatory movements (Wilson et al., 2004; Pulvermuller et al., 2006; Skipper et al., 2009; Mottonen et al., 2013). Further support that there is a relationship is provided by repetitive TMS studies in which disruptions to the PMC affects perception of speech sounds (Meister et al., 2007; D’Ausilio et al., 2009).

However, there is also evidence that speech perception involves some different brain areas to those used in production (Obleser and Eisner, 2009). Thus, perception is mainly associated with activation in the dorsolateral temporal cortexes (Obleser and Eisner, 2009). The involvement of other brain areas in speech perception in some studies may have resulted from additional task influences such as semantic processing (Davis and Johnsrude, 2003; Scott et al., 2006). There is also neuropsychological support for some independence of speech perception and production since impairments in speech perception can be dissociated from impairments in speech production in patients with brain lesions (Blank et al., 2003; Crinion et al., 2006).

Thus, it is unclear whether and how the speech motor areas are involved in the speech perception difficulties of PWS. The questions this study addressed were as follows: (1) Are there any functional activity differences between PWS and controls in the speech motor areas during speech perception, and if so, are these functional activity differences in the speech motor areas coincident across speech perception and production tasks? (2) After the motor areas that show different functional activity between PWS and controls in speech perception and production tasks were identified, their relationship with speech perception areas was compared between PWS and controls.

The following steps were performed to address the first question. (1) A speech perception task was used to identify functional activity differences associated with the speech perception difficulties of PWS, compared to controls. This task has been widely used to assess the ability of speech perception elsewhere in the literature (e.g., Chen et al., 2010; Klein et al., 2012; Krieger-Redwood et al., 2013). (2) A picture-naming task was employed to identify functional activity differences associated with the speech production difficulties of PWS, compared to controls. A picture-naming rather than a word-reading task was used to avoid influences of orthographic forms on phonological retrieval (Warren and Morton, 1982; Glaser, 1992). In this task, the names of the object pictures had different lengths, being three syllables long in the first naming condition (short name, SN) and 5–7 syllables long in the second naming condition (long name, LN). According to Levelt et al.’s (1999) theory, fundamental requirements of speech production are the retrieval, assembly and execution of syllable-sized motor programs. Thus, the computational load on the speech production process should vary with the number of syllables in the motor program (Lu et al., 2010a). The load-manipulation allowed the brain areas that are involved in the speech production process to be identified as those regions whose activity varied with word length (LN-SN). (3) After any functional activity differences associated with speech production and perception difficulties were identified separately in PWS compared to controls, a conjunction analysis was conducted to identify potential brain areas that showed functional activity differences between PWS and controls in both the speech production and perception tasks.

To address the second question, a Granger causality analysis (GCA) was performed. GCA is a method that uses vector autoregressive models to measure the causal relationship between time series such as the fMRI data collected here (Roebroeck et al., 2005). GCA has been employed to identify causal neural connection differences between patients and controls during resting state (Miao et al., 2011; Guo et al., 2014). GCA was applied to the resting-state fMRI data in the current study in order to exclude any potential confounding influence of tasks (Miao et al., 2011; Guo et al., 2014). Brain areas that were identified in the speech production task were selected as the target seeds, and then the bi-directional causal connections between the seeds and the whole brain areas were compared between PWS and controls. Significant causal connection differences might (indicating a relationship) or might not (indicating independent processing) appear in connections between the speech motor and speech perception areas between PWS and controls (e.g., IFC/insula/PMC to auditory temporal cortex).

## Materials and Methods

### Participants

Thirteen adult PWS (mean age = 23 ± 2.25 years) and 13 fluent controls (mean age = 24 ± 1.45 years) were recruited. All participants were male native Mandarin speakers. Interviews were conducted and no personal or family history of psychiatric, neurological or other disorders was reported except for stuttering in PWS. All participants were right-handed as assessed by the Edinburgh Handedness Inventory (Oldfield, 1971). The mean years of education for both groups were 17. There was no significant group difference in either age [*t*(24) = -0.258, *P* = 0.799] or educational years [*t*(24) = -0.867, *P* = 0.394] between PWS and controls. PWS were not involved in any treatment program and all of them reported that they had started to stutter before teenage.

The study was approved by the ethics committee of the State Key Laboratory of Cognitive Neuroscience and Learning, Beijing Normal University. Written informed consent was obtained from each participant.

### Assessment of Stuttering

Fluency of the participants was assessed using the Stuttering Severity Instrument version III (SSI-3) (Riley, 1994). Specifically, a spontaneous speech sample of at least 300-syllables and a reading of a standard 300-syllable text were video- recorded. Percentage of stuttered syllables (%SS) was computed by taking the number of disfluent syllables and dividing it by the total number of syllables and multiplying by 100 following the guidelines in Riley (1994). Any physical concomitants that occurred whilst the recordings were made (Riley, 1994) were noted independently by two research assistants. The %SS of PWS in conversation was ≥3%, and the SSI-3 severity scores were at least mild (mean = 31, *SD* = 4.88). Fluent controls were assessed in the same way and all met the criterion of disfluency scores <3%. No physical concomitants as assessed by SSI-3 were observed for the fluent controls. Each control also self-reported that they did not stutter.

### Experimental Tasks and Materials

#### Speech Perception Task

One hundred low frequency (<50 per million) two-character words were selected from the Corpus System of Modern Chinese^1^ database. A female Mandarin speaker was recorded as she spoke the words and these were stored as.WAV files. Half of the words were used for vowel judgment (VJ) condition, and the other half of the words were used for consonant judgment (CJ) condition.

During the VJ condition, the words were split into five blocks with words randomly assigned to blocks. For each block, visual instructions were presented for 5 s on the back-projection screen mentioned below. This indicated the target vowel that was to be judged. Then a string of stars (“^∗∗∗^”) was displayed for 500 ms, after which the task trials began. Each trial lasted 3 s. A trial began when a word was played to a participant through headphones. A fixation “+” was presented on the screen simultaneously with the word stimulus. The participants were required to attend to the fixation “+,” not to move their mouths, and judge the identity of the vowel on the second character of the word. For instance, the target vowel presented at the start could be /i/ or /ü/ for the 10 two-character words, examples of which are /bǐ jì/ (handwriting) and /miàn jù/ (mask). Five different pairs of target vowels were employed across the five blocks. Participants pressed a button beneath either the left or the right hand to register their decision about the target (i.e., whether the vowel was /i/ or /ü/). The correct responses when the identity of the vowel on the second character of the words given as examples earlier were judged, would be the /i/ (e.g., left button) for /bǐ jì/ and the /ü/ (e.g., right button) for /miàn jù/. The mapping of the vowels to response buttons was counterbalanced across participants. Behavioral reaction time (RT) data were acquired and used to establish whether there were speech perception difficulties in PWS. Previous evidence has shown that RT in such phonological perception tasks provides a useful measure that selectively targets speech perception in adults (Sucena et al., 2013). A 15-s rest interval was given between task blocks and the scanning data in this interval were used as a baseline in the imaging analysis. During the baseline period, a fixation “+” was presented on the screen. The participants were required to attend to the fixation “+” and not to move.

The arrangement for the CJ condition was the same as the VJ condition except that the participants judged the identity of the consonant on the second character of the word. For instance, the target consonant could be “b” or “p” in the 10 two-character words of a block such as /sōng bǎi/(pine) and /huà piàn/(picture). The full list of words and the target phonemes are given in **Supplementary Table S1**.

#### Speech Production Task

One hundred simple line drawings of common objects were selected from a standardized picture database (Zhang and Yang, 2003). Sixteen participants who were not involved in the scanning study assessed the pictures for consistency of names given, familiarity of concepts, and visual complexity. The names of half of the pictures were three syllables in length (SN) and the names of the other half were 5–7 syllables in length (LN).

Participants practiced the task before the experiment began. A 9-s interval occurred at the beginning of the experiment to allow the scanner to stabilize. Then, there were seven task-baseline alternating blocks. The length of the task period in these blocks was set at random to 42, 49, or 56 s, whereas the baseline period was fixed at 21 s in all blocks. During the task period of each block, each picture was presented on the screen for 1500 ms and this was followed by a blank screen for 3500 ms. When the pictures appeared on the screen, the participants were required to name the pictures aloud as quickly and accurately as possible. The voice responses were recorded digitally using an MRI-compatible microphone. Then, a “^∗∗∗^” string was presented on the screen for 2000 ms. During the baseline period of each block, a fixation “+” was presented on the screen. The participants were required to attend to the fixation “+” and not to move.

In the speech production task, verbal response duration (DU) rather than RT was used as the performance index because (1) results based on RT is not consistent in the stuttering literature: for example, whilst some studies reported difference between PWS and controls (Cross and Luper, 1983; Peters et al., 1989; Pellowski and Conture, 2005; Maxfield et al., 2015), others do not (Venkatagiri, 1982; Till et al., 1983; Harbison et al., 1989; Kelly and Conture, 1992; Arnold et al., 2005; Sasisekaran et al., 2006; Hennessey et al., 2008); (2) For longer utterances, DU reflects the accumulated effect of retrieval, assembly and execution of syllable-sized motor programs during speech production (Harbison et al., 1989; Maske-Cash and Curlee, 1995); (3) There is evidence indicating that DU of naming is sensitive to variables that influence pre-production processes because sub-lexical information about a word’s pronunciation can be used to initiate a naming response before the whole response is fully prepared.

However, as the DU data in the speech production task may be confounded by different subtypes of stuttering symptom within and across individuals (Jiang et al., 2012), %SS may be a more sensitive indicator of speech production performance in PWS. Thus, %SS was used as an index of speech production performance in PWS below, whereas the DU data was used to assess the effectiveness of the load-manipulation of the task design in both groups.

### FMRI Data Acquisition

Resting-state and task fMRI data were acquired from all participants on a Siemens TRIO 3T scanner at the MRI Center of Beijing Normal University. Participants lay supine within the MR scanner with their head stabilized by foam padding. An MRI-compatible headphone was used to reduce the perceived level of scanner noise and to present the auditory stimuli. A liquid-crystal projector displayed visual stimuli from inside the MR control room onto a back-projection screen located at the foot of the MR scanner. Participants viewed the stimuli via a mirror attached to the head coil above their eyes. E-prime software (v.1.2, Psychological Software Tools, Pittsburgh, PA, USA^2^) was used to present stimuli.

#### Resting-State Scan

The resting-state scan was always performed first. Participants were instructed to close their eyes, relax, and remain stationary. The axial gradient-recalled echo-planar images (EPI) were acquired in an 8-min task-free scan. The parameters were as follows: Repetition time (TR) = 2000 ms; echo time (TE) = 30 ms; flip angle = 90°; slice thickness = 4 mm; in-plane resolution = 3.1 mm ^∗^ 3.1 mm; number of interleaved slices = 33.

#### Structural Scan

Structural images were obtained with a high-resolution T1-weighted MP-RAGE sequence: TR = 2530 ms; TE = 3.30 ms; flip angle = 7°; slice thickness = 1.3 mm; in-plane resolution = 1.3 mm ^∗^ 1.0 mm; number of interleaved sagittal slices = 128.

#### Speech Perception Task Scan

TR = 3000 ms (delay = 1500 ms); TE = 30 ms; flip angle = 90°; field of view = 200 mm; matrix = 64 × 64; slice thickness = 5 mm; in-plane resolution = 3.1 mm × 3.1 mm; number of interleaved axial slices = 25.

#### Speech Production Task Scan

TR = 7000 ms (delay = 5000 ms); TE = 30 ms; flip angle = 90°; field of view = 200 mm; matrix = 64 × 64; slice thickness = 5 mm; in-plane resolution = 3.1 mm × 3.1 mm; number of interleaved axial slices = 33.

### FMRI Data Analysis

#### Preprocessing of the Data

Data preprocessing and statistical analyses were conducted using the standard parameters of the Statistical Parametric Mapping package (SPM8, Wellcome Trust Center for Neuroimaging, London, UK). The first two volumes of each participant’s functional images were discarded prior to data analysis. During preprocessing, the functional images were slice-time corrected and realigned. During spatial normalization, the functional images were co-registered to high-resolution T1 images at individual participant level. The images were then spatially normalized to the Montreal Neurological Institute (MNI) template (spatial resolution = 2 mm × 2 mm × 2 mm) by using unified segmentation T1 images (Ashburner and Friston, 2005). Finally, the images were smoothed using a 6-mm full-width at half-maximum Gaussian filter. The resting-state data were further band-pass filtered between 0.01 and 0.08 Hz.

#### Statistical Analysis of the Task FMRI Data

##### First-level analysis

For the data in each condition of each task, the contrast of interest was estimated using a general linear model (GLM). The visual instructions and the string of stars (“^∗∗∗^”) were modeled together, but separately for VJ and CJ in the speech perception task scan. This ensured the temporal specificity of the response to the VJ/CJ stimuli as only the orthogonal regressor components were taken into account (Poline et al., 2007). The conditions of interest were modeled using a boxcar function with the respective duration convolved with a canonical hemodynamic response function. Data were corrected for serial autocorrelations. The same procedures were applied with the speech production task (i.e., LN and SN). Finally, for the speech perception task, the contrasts of interest were each of the conditions (i.e., VJ and CJ) relative to their specific baselines. For the speech production task, the contrasts of interest were each of the conditions (i.e., LN and SN) relative to their specific baselines, as well as LN relative to SN (i.e., LN–SN). As the load-manipulation aimed to identify the brain areas that are involved in the speech production process (i.e., those regions’ activity varied with word length), the LN–SN contrast is presented in the main text, whereas the contrasts of LN and SN to their baseline are reported in the **Supplementary Figures S1** and **S2**. The data were high-pass filtered with a cutoff frequency of 128 Hz. The movement parameters derived from the realignment stage were included in the GLM as nuisance variables.

##### Second-level random-effect analysis

First, *t*-tests were conducted on the speech perception (VJ and CJ as separate conditions) and production (LN–SN) tasks to establish any group differences between PWS and controls. Significance was determined using joint expected probability distribution with height and extent thresholds *P* < 0.05 implemented with 3dClustSim^3^ (Height: *P* < 0.005; Extent: cluster > 398 mm^3^) (Poline et al., 1997). Second, a conjunction analysis was conducted to establish coincidence of functional activity differences between PWS and controls in the speech perception and production tasks. The contrasts involving PWS minus fluent controls in VJ, CJ, and LN–SN were used. The Conjunction Null hypothesis was assessed, and an intersection SPM or ‘minimum T-field’ was computed (corrected, *P* < 0.05). The *P*-value refers to the threshold of the conjunction. Note that the minimum *T*-values do not have the usual Student’s T-distribution and small minimum *T*-values can be highly significant. This analysis can be thought of as enabling an inference that all contrasts between PWS and controls (i.e., VJ, CJ, and LN–SN) showed group differences.

#### GCA on the Resting-State Data

Granger causality analysis was applied to the resting-state fMRI data. First, GCA was performed for each participant using the Resting-State fMRI Data Analysis Toolkit - GCA (REST-GCA) by a seed-to-voxel approach (Song et al., 2011; Zang et al., 2012). Specifically, the brain areas identified in the speech production task were used as the target seeds, and the signed-path coefficients were computed (Chen et al., 2009). The coefficients represented bi-directional causal connections between the seeds and the whole-brain voxels in resting state (i.e., F_x->__y_ and F_y-__>__x_) (Roebroeck et al., 2005). The order was one. For simplicity, no covariate was used. Second, two-sample *t*-tests were conducted in a second-level random-effect analysis to compare the causal connections (i.e., the connections from/to the target seed region to the rest of the brain) between PWS and controls (*P* < 0.05, corrected by joint expected probability distribution with height and extent thresholds).

#### Correlations between Causal Connections and Speech Perception Performance

To establish the relationship between the resting-state causal connections and behavioral performance, partial correlation analysis was conducted between the strength of causal connection and RT data in VJ while controlling for the influence of CJ. A similar analysis was conducted on CJ where the influence of VJ was controlled.

A Hierarchical Linear Modeling analysis was conducted to examine whether the relationship between the functional connection and speech perception performance was modulated by speech production performance, Specifically, in a linear regression procedure, the behavioral performance in speech production was entered into the model first, and RT in VJ and CJ were entered next. The *R^2^ change* reflects whether the contribution of the RT data to the model was significant after controlling for the effect of behavioral performance in speech production.

## Results

### Confirmation of Speech Perception and Production Difficulties in PWS

**FIGURE 1:**
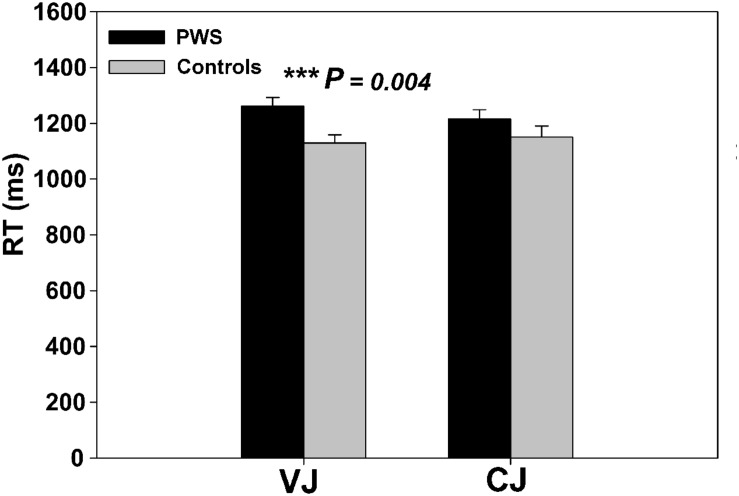
**Behavioral response time in VJ and CJ for different groups**.

Independent two-sample *t*-tests on the behavioral RT data from the speech perception task showed that PWS had significantly longer RTs than controls in VJ [*t*(24) = 3.136, *P* = 0.004], but not in CJ [*t*(24) = 1.277, *P* = 0.214] (**Figure 1**). The VJ finding confirmed the speech perception difficulties in PWS specifically during vowel perception.

An ANOVA on DU from the speech production task found a significant naming condition effect [*F*(1,24) = 344.09, *P* < 0.001] and interaction effect between condition and group [*F*(1,24) = 6.86, *P* = 0.015], whereas the group main effect was only marginally significant [*F*(1,24) = 3.72, *P* = 0.066]. Simple effect analyses indicated a significant group difference in LN only (*P* = 0.032). These results confirmed the current hypothesis that the computational load on the speech production process varied with the number of syllables in the motor program (Lu et al., 2010a), and that PWS have different response to the syllable-length manipulation to fluent controls.

In addition, the mean %SS as described in the assessment of speech fluency was 12% (*SD* = 1.98) for PWS and 0% (*SD* = 0.01) for controls. PWS and controls differed significantly in %SS [*t*(24) = 21.104, *P* < 0.001], which further confirmed the speech production difficulties of PWS. %SS was used as an index of speech production performance below.

### Functional Activity Difference between PWS and Controls during Speech Perception

**FIGURE 2:**
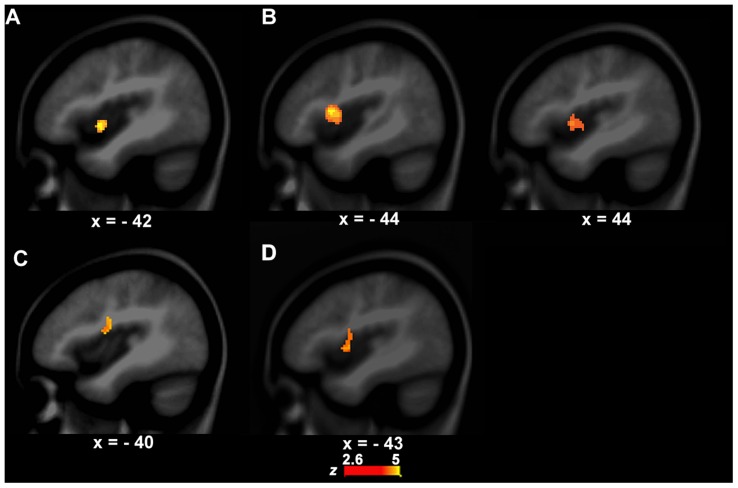
**Functional activity differences between PWS and controls. (A,B)** show functional activity differences between PWS and controls in the speech perception task (left, **A**, VJ, right, **B**, CJ). **(C)** Shows functional activity differences between PWS and controls in the speech production task (i.e., LN–SN). **(D)** Shows conjunction results across the three group contrasts (i.e., between PWS and controls in VJ, CJ, and LN–SN).

The left anterior insula that is involved in speech motor control (Dronkers, 1996; Baldo et al., 2011) showed a significant group difference in functional activity between PWS and controls during VJ. That is, the left anterior insula (BA13, *x, y, z* = -42, 2, -4, *z* = 4.09, cluster size = 408 mm^3^) had stronger activity in PWS than in controls (**Figure 2A**). There were no significant group differences in VJ in any auditory temporal cortical areas.

Although the CJ’s behavioral data did not differ significantly between PWS and controls, the brain activity in the left (BA13, *x, y, z* = -44, 4, 2, *z* = 3.10, cluster size = 462 mm^3^) and right anterior insula (BA13, *x, y, z* = 44, 10, 10, *z* = 5.01, cluster size = 2256 mm^3^) was significantly stronger in PWS than in controls (**Figure 2B**). Again, no auditory temporal cortical areas showed significant group difference in CJ.

### Functional Activity Difference between PWS and Controls during Speech Production

The left IFC/anterior insula (BA44/13, *x, y, z* = -40, -4, 18, *z* = 3.01, cluster size = 456 mm^3^) showed a significant difference in functional activity between PWS and controls across conditions when the computational load on speech production varied (i.e., LN–SN) (**Figure 2C**). Further *post hoc* analysis showed that this area had significantly stronger activity in LN than in SN (*P* = 0.012) in controls, but not in PWS (*P* = 0.812), indicating a reduction in the function of this area during speech motor control in PWS.

### Coincident Functional Activity Difference between PWS and Controls during Speech Production and Perception

The conjunction analysis showed that the left IFC/anterior insula (BA44/13, *x, y, z* = -43, 0, 5, *z* = 4.23, cluster size = 1472 mm^3^) has significant functional activity differences between PWS and controls in both the speech perception (VJ and CJ) and production (LN–SN) tasks (**Figure 2D**).

### Causal Connection Differences between PWS and Controls

#### Connections from the left IFC/Anterior Insula to other Brain Areas

**FIGURE 3:**
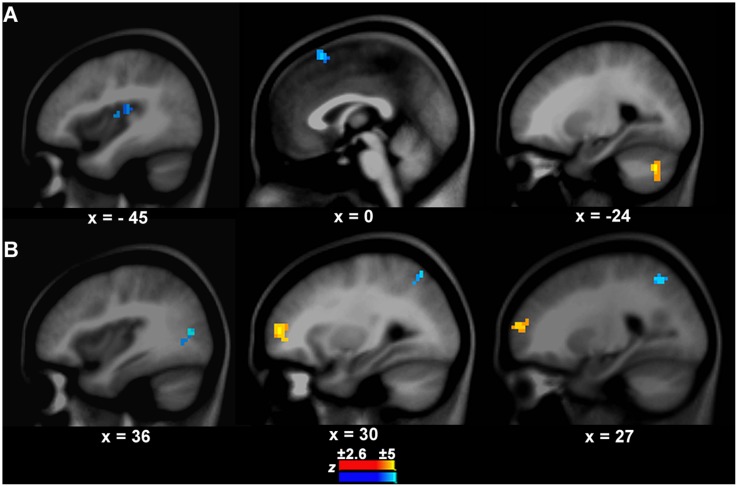
**Connection differences between PWS and controls from the left IFC/anterior insula to other brain areas **(A)** and vice versa **(B)****. The yellow and blue colors indicate stronger or weaker connections respectively in PWS than in controls.

There was a significant difference in the neural activity of the left IFC/anterior insula between PWS and controls during speech production. Thus, this area was selected as the target seed. The GCA results showed that the causal connection from the seed to the auditory temporal cortical area, i.e., left Heschl’s gyrus (BA41, *x, y, z* = -45, -24, 12, *z* = -3.10, cluster size = 570 mm^3^), was weaker in PWS than in controls (**Figure 3A**). Additionally, there was also a weaker causal connection from the seed to the pre-supplementary motor area (preSMA, BA6, *x, y, z* = 0, 18, 57, *z* = -3.61, cluster size = 733 mm^3^), but a stronger connection to the left cerebellum (Crus1, *x, y, z* = -24, -69, -36, *z* = 4.49, cluster size = 760 mm^3^) in PWS than in controls (**Figure 3A**).

#### Connections from other Brain Areas to the Left IFC/Anterior Insula

The connection from the right middle frontal cortex (BA10, *x, y, z* = 30, 54, 6, *z* = 3.41, cluster = 1358 mm^3^) to the seed was stronger in PWS than in controls (**Figure 3B**). In contrast, the connections from the right middle occipital cortex (BA19, *x, y, z* = 36, -75, 12, *z* = -3.39, cluster size = 679 mm^3^) and superior parietal cortex (BA7, *x, y, z* = 27, -57, 48, *z* = -3.01, cluster size = 570 mm^3^) to the seed were weaker in PWS than in controls (**Figure 3B**). No differences were found in the connections from the auditory temporal cortical areas to the seed between PWS and controls.

### Correlations between Strength of Causal Connections and Speech Perception Performance

**FIGURE 4:**
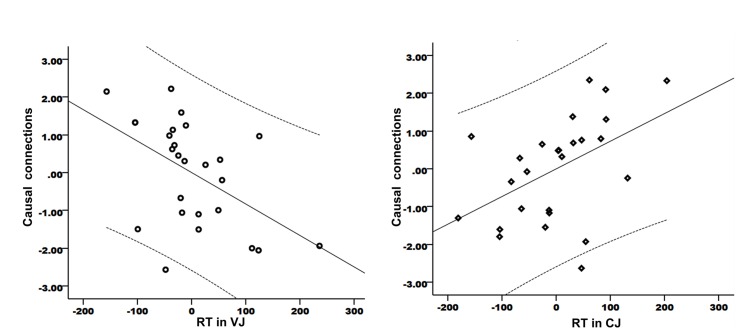
**Partial correlations between the strength of the causal connection from the left IFC/anterior insula to Heschl’s gyrus and the RT data in VJ **(A)** and CJ **(B)****. The curves indicate 95% confidence interval. The *x*- and *y*-axes have been normally scaled.

The focus of this study was the relationship between speech production and speech perception. Therefore, the causal connection from the left IFC/anterior insula to left Heschl’s gyrus was of particularly interest. As this connection showed significant group difference between PWS and controls in resting state, it is important to establish whether the strength of this connection correlated with speech perception performance within the patient group. The results showed significant partial correlations between the strength of this connection and the RT data in VJ (*r* = -0.5, *P* = 0.011) (**Figure 4A**) and CJ (*r* = 0.472, *P* = 0.017) (**Figure 4B**, the axes were normalized). This result indicated that the connection from the speech motor area to the speech perception area played a role in speech perception.

More importantly, there were significant correlations between the strength of the connection and %SS (*r* = 0.593, *P* = 0.032) and between %SS and RT in VJ (*r* = 0.606, *P* = 0.037). In a Hierarchical Linear Model analysis, no significant correlations were found between the strength of this connection and the RT data in VJ (*R^2^* change = 0.052, *P* = 0.37) when %SS were controlled for. However, when the RT data in VJ were controlled for, there was a still significant correlation between the connection’s strength and %SS (*R^2^* change = 0.402, *P* = 0.027). This finding further indicated that the relationship of the connection between the speech motor area and the auditory areas with the speech perception performance was modulated by speech production performance.

## Discussion

This study examined whether functional activity difference between PWS and controls in speech perception is independent of, or related to, that in speech production. Based on the point of view that there is a production-perception relationship, it was hypothesized that anomalous neural activity in the speech motor area such as the left IFC, anterior insula and PMC would be involved in any speech perception difficulties of PWS. The data from the speech perception task revealed that neural activity in the left anterior insula was stronger in PWS than in controls. A conjunction analysis that included the speech perception and production tasks revealed coincident neural activity difference between PWS and controls in a speech motor area (i.e., left IFC/anterior insula) covering the left pars opercularis (BA44), anterior insula (BA13), and a small part of the Rolandic operculum (BA43). This area plays a critical role in speech articulation, particularly for intra- and inter-syllabic coordination of complex articulatory movements (Dronkers, 1996; Baldo et al., 2011). The current results were consistent with previous high-density ERP (Liotti et al., 2010) and fMRI evidence (Chang et al., 2009) on PWS that reported neural activity differences in the speech motor areas between PWS and controls. The present findings supported the hypothesis that anomalous neural activity in the speech motor area is involved in both the speech production and perception difficulties of PWS.

The left IFC/anterior insula showed increased neural activity when the condition changed from SN to LN in the controls, but not in PWS. Anomalous neural activity in this area or connectivity between this area and the motor and auditory areas have been reported previously in various speech production tasks in adult PWS (Lu et al., 2009, 2010a,b; Jiang et al., 2012; Kell, 2014). Most importantly, the current results indicated that this area failed to respond to the manipulation of computational load in the speech production task in PWS. This suggests that the difference in the neural activity of this area between PWS and controls reflects lower functionality of this area in PWS during speech motor control.

The lower functionality in the left IFC/anterior insula may have an impact on the auditory area, which would affect speech perception. This possibility | was supported by the GCA results. The connectivity pattern in the GCA results showed that in resting state the causal connection from the left IFC/anterior insula to left Heschl’s gyrus was weaker in PWS than in controls. Moreover, the strength of this causal connection correlated significantly with speech perception performance. After controlling for speech fluency level, the correlations between the strength of this connection and speech perception performance was not significant, which further suggested an influence of speech production performance on speech perception performance. These results help to integrate previous evidence about speech perception difficulties in PWS. Specifically, Liotti et al. (2010) in a high-density ERP study identified abnormal activity in scalp-recorded electrical potentials in the first (20–80 ms) and third (225–375 ms) negative peaks (N1 and N3) during speech perception. The source of N1 was located in the right PMC, whereas N3 was located in the right secondary auditory cortex. In an fMRI study Chang et al. (2009) found widely distributed anomalous activity across motor and auditory cortexes during perception of both speech and non-speech stimuli in PWS compared to controls. Taking the high temporal resolution ERP evidence and the high spatial resolution fMRI evidence together, it seems that (a) the speech motor area is involved in speech perception (Chang et al., 2009; Liotti et al., 2010), and (b) the speech motor area is activated earlier than the auditory area during the speech perception process (Liotti et al., 2010). The current results further suggest that the later-activated auditory area receives inputs from the speech motor area. Thus, if the functionality of the speech motor area is reduced in PWS, processing in the auditory temporal area might be affected. One possibility is that the functionally anomalous left IFC/anterior insula fails to provide accurate articulatory gestural information to the auditory temporal cortex in PWS (Liberman and Whalen, 2000), resulting in reduced connectivity from the speech motor area to the auditory area.

In addition, altered causal connections in the PWS were also found from the left IFC/anterior insula to the preSMA and cerebellum, and from the right occipital, parietal, and frontal areas to the left IFC/anterior insula. These brain areas cover widely distributed neural networks that are responsible for visual, auditory, motoric, and attentional control. Previous studies on both PWS and CWS have reported anomalous resting-state functional connectivity between the left IFC and other speech areas (Lu et al., 2012; Chang and Zhu, 2013). As the resting-state functional connectivity reflects the intrinsic functional architecture of the human brain (Biswal et al., 1995; Cole et al., 2014; Sripada et al., 2014), it seems reasonable to conclude, based on these findings, that the intrinsic functional architecture of the brain is altered in PWS compared to controls.

Although PWS did not differ behaviorally from controls in consonant perception in this study, they still showed a neural activity difference from controls in the left anterior insula with these stimuli. Moreover, PWS additionally recruited the right homologous area to the left anterior insula in consonant perception but not in vowel perception. One possibility is that the right anterior insula plays a compensatory role in CJ because of the anomaly of the left anterior insula, and this compensation was successful so that PWS did not differ behaviorally from controls. However, In VJ, PWS did not have this compensatory mechanism and thus showed a behavioral deficit. Therefore, the neural activity difference between PWS and controls in CJ may reflect a compensatory mechanism. This speculation was further supported by the opposite patterns of correlations between causal connection from the left IFC/anterior insula to the Heschl’s gyrus and performance in different speech perception conditions. That is, the better the performance (i.e., shorter RT) in VJ, the stronger the causal connection (i.e., closer to controls); the better the performance in CJ, the weaker the connection (i.e., closer to PWS) in CJ. Another possibility is that this difference is a reflection of neural processing differences between vowels and consonants whereby, compared with vowel processing, consonant processing requires more involvement of the right frontal cortex (Carreiras and Price, 2008). Thus, the recruitment of the right in addition to the left anterior insula may be a distinct feature of consonant processing. This interpretation, however, makes it difficult to assess whether the behavioral performance and brain activity in CJ were related or independent.

The data from the speech perception task did not reveal regional neural activity difference between PWS and controls in the auditory cortex. It is likely that the sensory processing in the auditory area of PWS is intact. There is evidence from adults with dyslexia that this applies (Boets et al., 2013); however, the functional and structural connectivity between the auditory areas and the left IFC is weakened in these patients, suggesting impaired access to the intact phonetic representations (Boets et al., 2013). Thus, it is also possible that PWS have difficulties in accessing the articulatory gestures during coding of sensory inputs, rather than in processing the sensory inputs themselves.

## Conclusion

The present results provide insights into the debates about the relationship between speech perception and production. Specifically, whilst speech motor areas have been reported to be active during speech perception (Wilson et al., 2004; Pulvermuller et al., 2006; Skipper et al., 2009; Mottonen et al., 2013), the activation has been attributed to influences of other factors such as semantics (Davis and Johnsrude, 2003; Scott et al., 2006). However, the present results showed a causal connection between speech motor and auditory areas even in resting-state where no tasks were performed, and this production-perception connection correlated significantly with speech perception performance. Thus, the present findings suggest that the involvement of the speech motor area in speech perception may not be entirely a result of the confounding task factors. Moreover, the present findings suggested that although PWS have difficulties in both speech production and perception, their difficulties in speech perception may be impacted by their difficulties in speech production.

## Author Contributions

CL designed the experiment, analyzed the data, and wrote the paper. YL, LZ, and GS collected and analyzed the data. LL and GD designed the experiment; PH designed the experiment, and wrote the paper.

## Conflict of Interest Statement

The authors declare that the research was conducted in the absence of any commercial or financial relationships that could be construed as a potential conflict of interest.

## Footnotes

1 http://www.cncorpus.org

2 www.pstnet.com

3 afni.nimh.nih.gov

## Supplementary Material

FIGURE S1 **Task-induced brain activations during SN and LN in each individual group**. Only positive activations were reported here.

FIGURE S2 **Contrast of LN-SN in each individual group**. Only positive activations were reported here.

Table S1 **Full list of the experimental materials in the speech perception tasks**.
