# Supplementary figures and images for: Relationship between Speech Production and Perception in People Who Stutter
Source: Front Hum Neurosci. 2016 May 18;10:224. doi: 10.3389/fnhum.2016.00224 (PMC4870257; doi:10.3389/fnhum.2016.00224)

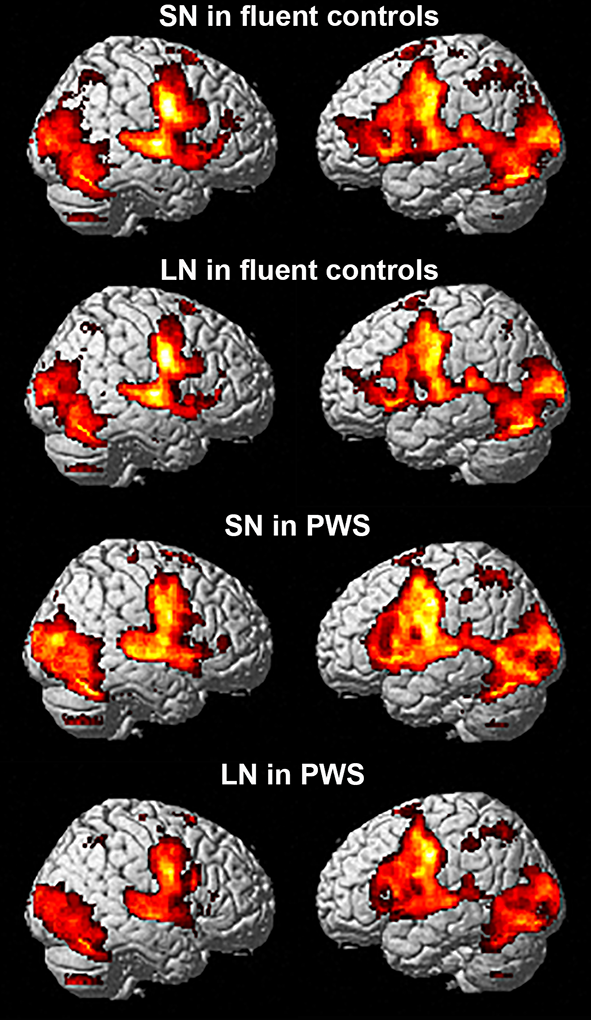

Supplement: FIGURE S1 — Task-induced brain activations during SN and LN in each individual group. Only positive activations were reported here. [file Image_1.TIF]

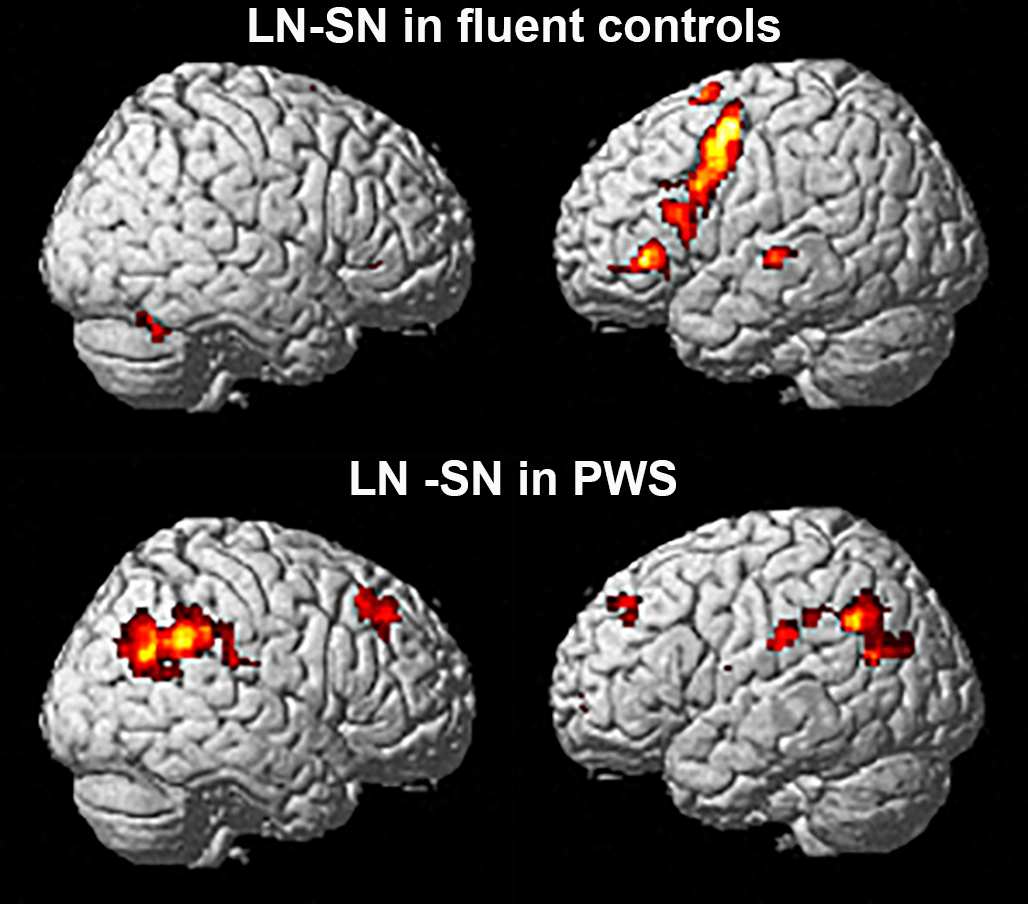

Supplement: Supplementary file 2 [file Image_2.TIF]
