# Supplementary material for: Relationship between Speech Production and Perception in People Who Stutter
Source: Front Hum Neurosci. 2016 May 18;10:224. doi: 10.3389/fnhum.2016.00224 (PMC4870257; doi:10.3389/fnhum.2016.00224)
Supplement: Table S1 — Full list of the experimental materials in the speech perception tasks. [file Table_1.DOCX]

**Table S1 Full list of the experimental materials in the speech perception tasks**

| **Block** | **First character** | **Second character** | **Target vowel** | **First character** | **Second character** | **Target consonant** |
| --- | --- | --- | --- | --- | --- | --- |
| 1 | bǐ | jì | ì | lín | bā | b |
| 1 | zhé | lǐ | ǐ | gǔ | bǎn | b |
| 1 | fèi | qì | ì | huán | bǎo | b |
| 1 | jiān | xì | ì | xī | bié | b |
| 1 | qíng | yì | ì | dǐng | bù | b |
| 1 | miàn | jù | ù | péng | dà | d |
| 1 | qíng | lǚ | ǚ | gū | dān | d |
| 1 | jìn | qǔ | ǔ | chàng | dǎo | d |
| 1 | zàn | xǔ | ǔ | shé | dié | d |
| 1 | shù | yǔ | ǔ | mù | dǔ | d |
| 2 | piāo | bó | ó | wán | kàng | k |
| 2 | fēng | bō | ō | suí | kǒu | k |
| 2 | tú | mò | ò | lěng | kù | k |
| 2 | zāo | pò | ò | péi | kuǎn | k |
| 2 | wàng | wǒ | ǒ | duō | kuī | k |
| 2 | pǐn | dé | é | gǔn | tàng | t |
| 2 | dān | gē | ē | xiǎo | tōu | t |
| 2 | xiān | hè | è | qīng | tǔ | t |
| 2 | qǐng | kè | è | qì | tuán | t |
| 2 | kuáng | rè | è | pán | tuǐ | t |
| 3 | hòu | dào | ào | sōng | bǎi | b |
| 3 | chuō | hào | ào | tóu | bēn | b |
| 3 | tú | láo | áo | mì | bì | b |
| 3 | fàng | shào | ào | tū | biàn | b |
| 3 | fǎng | zào | ào | biàn | bó | b |
| 3 | yān | dòu | òu | zhèng | pai | p |
| 3 | wèn | hòu | òu | miàn | pén | p |
| 3 | chǒu | lòu | òu | cǎo | pí | p |
| 3 | chū | shòu | òu | huà | piàn | p |
| 3 | bēn | zǒu | ǒu | ā | pó | p |
| 4 | mǎ | guà | uà | gū | jì | j |
| 4 | yuán | huá | uá | hào | jiǎo | j |
| 4 | cū | huà | uà | xiōng | jīn | j |
| 4 | xì | shuǎ | uǎ | jìng | jiǔ | j |
| 4 | fěn | shuā | uā | huà | jù | j |
| 4 | kāi | guó | uó | diu1 | qì | q |
| 4 | dìng | huò | uò | pèng | qiǎo | q |
| 4 | téng | luó | uó | chéng | qīn | q |
| 4 | fēng | shuò | uò | qǐ | qiú | q |
| 4 | lè | suǒ | uǒ | shè | qǔ | q |
| 5 | fěn | chén | én | zhǐ | nán | n |
| 5 | héng | gèn | èn | wū | ní | n |
| 5 | bā | hén | én | cuì | niǎo | n |
| 5 | jiāo | nèn | èn | zhí | niù | n |
| 5 | miàn | pén | én | wán | nòng | n |
| 5 | chì | chéng | éng | xuàn | làn | l |
| 5 | tián | gěng | ěng | líng | lì | l |
| 5 | mán | héng | éng | zuò | liào | l |
| 5 | xiào | néng | éng | zhēng | liú | l |
| 5 | chuī | pěng | ěng | lā | lǒng | l |
